# A porcine model system of BRCA1 driven breast cancer

**DOI:** 10.3389/fgene.2015.00269

**Published:** 2015-08-25

**Authors:** Howard Donninger, Katharine Hobbing, M. L. Schmidt, Eric Walters, Laurie Rund, Larry Schook, Geoffrey J. Clark

**Affiliations:** ^1^Department of Medicine, James Graham Brown Cancer Center, University of LouisvilleLouisville, KY, USA; ^2^Department of Pharmacology and Toxicology, James Graham Brown Cancer Center, University of LouisvilleLouisville, KY, USA; ^3^Department of Biochemistry, University of LouisvilleLouisville, KY, USA; ^4^Division of Animal Sciences, National Swine Resource and Research Center, University of MissouriColumbia, MO, USA; ^5^Department of Animal Sciences, University of Illinois at Urbana-ChampaignUrbana, IL, USA

**Keywords:** breast cancer, BRCA1, miRNA, SV40 LT, transformation

## Abstract

BRCA1 is a breast and ovarian tumor suppressor. Hereditary mutations in BRCA1 result in a predisposition to breast cancer, and BRCA1 expression is down-regulated in ~30% of sporadic cases. The function of BRCA1 remains poorly understood, but it appears to play an important role in DNA repair and the maintenance of genetic stability. Mouse models of BRCA1 deficiency have been developed in an attempt to understand the role of the gene *in vivo*. However, the subtle nature of BRCA1 function and the well-known discrepancies between human and murine breast cancer biology and genetics may limit the utility of mouse systems in defining the function of BRCA1 in cancer and validating the development of novel therapeutics for breast cancer. In contrast to mice, pig biological systems, and cancer genetics appear to more closely resemble their human counterparts. To determine if BRCA1 inactivation in pig cells promotes their transformation and may serve as a model for the human disease, we developed an immortalized porcine breast cell line and stably inactivated BRCA1 using miRNA. The cell line developed characteristics of breast cancer stem cells and exhibited a transformed phenotype. These results validate the concept of using pigs as a model to study BRCA1 defects in breast cancer and establish the first porcine breast tumor cell line.

## Introduction

Breast cancer is a leading cause of death in women and is one of the most common cancers in the world today. Up to 40,000 women are expected to die of breast cancer annually in the US alone (Siegel et al., 2011). The underlying causes of breast cancer development remain very much under investigation, but we now know that the BRCA1 tumor suppressor gene plays an important role in many breast cancers. Women who carry a BRCA1 germ line mutation have a cumulative lifetime risk of 50–85% of developing breast cancer (King et al., 2003). Although somatic BRCA1 mutations are rare in sporadic breast cancer, BRCA1 expression is down-regulated in ~30% of sporadic cases by allele loss or epigenetic mechanisms (Welcsh and King, 2001; Yang et al., 2001).

The function of BRCA1 remains poorly understood. It has a ubiquitin ligase activity and can control the stability/activity of proteins such as Claspin (Sato et al., 2012) and estrogen receptor alpha (Savage and Harkin, 2015). It is also a key player in modulating DNA repair (Zhang and Powell), replication fork stability (Pathania et al., 2011), senescence (Tu et al., 2013), oxidative stress (Marks, 2013), genomic stability (Savage and Harkin, 2015), and checkpoint induced cell cycle arrest (Huen et al., 2010). The complex role of BRCA1 in cellular homeostasis has made elucidating its key functions in cancer difficult.

Mouse models of BRCA1 deficiency have been developed in an attempt to understand the role of the gene *in vivo* (Ma et al., 2010). Although BRCA1 knockout provokes embryonic lethality in mice, conditional knockout of BRCA1 in breast tissue leads to tumor development after a long latency. The latency period can be strongly reduced by introducing defects in the p53 tumor suppressor to the animal system. These animal models have allowed the validation of therapies designed against BRCA1 defective tumors. However, even therapeutic approaches that were effective resulted in the emergence of resistant tumors (Ma et al., 2010). Further studies to examine approaches to overcome the resistance are limited by the short lifespan of the mice. Moreover, the subtle nature of BRCA1 function and the well-known discrepancies between human and murine breast biology (Dine and Deng, 2013) and cancer genetics (Kendall et al., 2005) may limit the utility of mouse systems in defining the function of BRCA1 in human cancer.

In contrast to mice, pigs exhibit very similar cancer genetics to humans (Adam et al., 2007). Moreover, their physiology and biochemistry is similar (Swindle et al., 2012) and their lifespan extends for decades. Consequently, a porcine model for breast cancer could prove a powerful tool for validating breast cancer therapies, preventative strategies and the clinical response to the emergence of drug resistance.

In order to validate the use of porcine systems in breast cancer research, we generated an immortalized porcine breast cell line using the SV40 LT oncoprotein (Chen and Hahn, 2003). We then used BRCA1 miRNA to generate a stable matched pair of cell lines that are positive or negative for BRCA1 expression. Characterization of the cells showed that BRCA1 knockdown induced enhanced growth and induced a transformed phenotype on the cells. Moreover, the transformed cells expressed markers characteristic of cancer stem cells. These results establish the first porcine breast cancer cell line and validate the concept of using porcine systems as a model to study BRCA1 defects in breast cancer.

## Materials and methods

### Porcine cell lines and transfections

Primary porcine breast epithelia cells were isolated as described in Prather et al. (1999) using a protocol approved by the IACUC of the University of Missouri-Columbia, Columbia, Missouri. They were transfected with pbabe puro SV40LT (Addgene #13970) using Lipofectamine 2000 (Invitrogen, Carlsbad CA) according to the manufacturer's instructions. Cells were selected in puromycin (Sigma, St Louis, MO) at 1 μg/ml. miRNA sequences corresponding to two different regions of porcine BRCA1 were designed using the Block-iT™ RNAi Designer (Invitrogen). Two single-stranded DNA oligonucleotides were designed for each sequence, one encoding the target pre-miRNA (top strand) and the other, its compliment (bottom). Each oligonucleotide also contained five nucleotides (TGCTG) derived from the endogenous miR-155 at the 5′ end and 19 nucleotides derived from miR-155 to form a terminal loop. The sequences of the two different oligo sets are as follows: #1 Top: 5′-TGCTGA TTGTTTGCAAACTGCAATCCGTTTTGGCCACTGACTGACGGATTGCATTGCAAACA AT-3′, #1 Bottom: 5′-CCT GATTGTTTGCAATGCAATCCGTCAGTCAGTGGCCAAAACGGATTGCAGTTTGCAAACAA TC-3′; #2 Top: TGCTGTATTAAAGCACCATGAGGGTCGTTTTGGCCACTGACTGACGACCCTCAGTGCTTT AATA-3′; #2 Bottom: 5′-CCT GTATTAAAGCACTGAGGGTCGTCAGTCAGTGGCCAAAACGACCCTCATGGTGCTTTA ATAC-3′.

The corresponding single-stranded oligos were annealed to generate a double-stranded oligo which was then cloned into the pcDNA™ 6.2-GW/EmGFP-miR vector (Invitrogen). Generation of the double-stranded oligos and cloning into the expression vector were performed using the BLOCK-iT™ Pol II miR RNAi Expression Vector Kit (Invitrogen) as described by the manufacturer. Stable transfectants were generated by transfecting the transformed pig mammary epithelial cells with 2 μg of the two different miRNA expression vectors, as well as a negative control consisting of a miRNA to LacZ, using Lipofectamine 2000 according to the manufacturer's instructions and selecting with Blasticidin (4 μg/ml).

### qRT-PCR

qRT-PCR was performed on total RNA isolated from the cells with Trizol using an iCycler Real-Time Detection System (Bio-Rad Laboratories, Inc., Hercules, CA) with the Quantitect SYBR Green RT-PCR Kit (Qiagen, Inc., Valencia, CA) as per the manufacturer's instructions. The fold change for each gene was calculated using the 2^−ΔΔCT^ method (Livak and Schmittgen, 2001) with GAPDH as the reference gene. The primers used were BRCA1 For: 5′-GTCCAAAGCGAGC AAGAGAA -3′, BRCA1 Rev: 5′- ACAGAAG CCCCACAGAGGA -3′; GAPDH For: 5′- CGATGC TGGTGCTGAGTATG- 3′, GAPDH Rev: 5′- GAAGGG GCAGAGATGATGAC- 3′.

### Western blots

Total cell lysates were prepared by lysing the cells in modified RIPA buffer (150 mM NaCl, 50 mM Tris, pH 7.5, 1% NP-40) supplemented with 100 μg/ml leupeptin, 100 μg/ml aprotinin and 1 mM sodium orthovanadate. BRCA1 and ALDH1 antibodies were obtained from Santa Cruz Biotechnology, Santa Cruz, CA., Actin antibodies were from Sigma (St. Louis MO) and EpCAM antibodies were from AbCam. HRP conjugated Trueblot secondary antibodies were purchased from eBioscience (eBioscience Inc. San Diego, CA) and western blots were developed using a Pierce ECL detection system (Thermo Scientific, Rockford IL).

### Growth curves

2 × 10^4^ cells/well were plated in six-well plates in normal growth medium and incubated for 6 days. Cell number was determined each day by counting the number of viable cells. Experiments were performed twice in duplicate.

### Matrigel

Fifty micro liters of Matrigel (BD Biosciences, San Jose, CA) was plated in a 96 well plate and allowed to set. Cells were trypsinized, washed in growth medium and plated at 5000 cells per well in 100 μl of growth medium. Hundred micro liters of medium +4% Matrigel was added and the medium changed every 4 days.

### Soft agar

Six well plates were prepared with 2 ml bottom agar (16 ml 1.8% molten Difco Bacto agar cooled to 42°C and mixed with 1.6 ml serum, 1.6 ml 10X PBS and mixed with 30.3 ml DMEM) and allowed to set. Cells were trypsinized, washed, and 3 × 10^4^ cells suspended in 1.5 ml growth medium. Three milli liters of liquid bottom agar was added to the cell dilution and 1.5 ml aliquoted into each well to set.

### Anoikis

Twelve well plates were treated with polyHEMA (Sigma) and allowed to dry overnight. 1 × 10^6^ cells were plated in each well and the cell viability measured after 48 h by trypan blue exclusion.

## Results

### Generation of an immortalized porcine epithelial cell line

**Figure 1:**
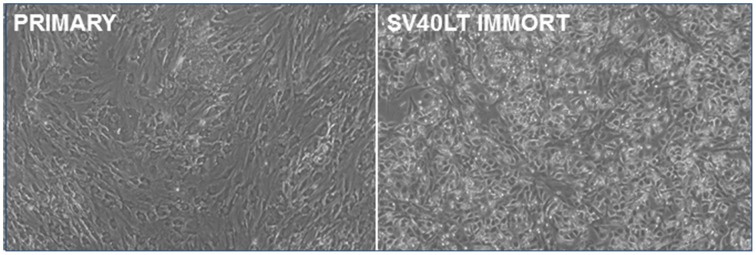
**Immortalization of pig mammary epithelial cells**. Primary pig breast epithelial cells were stably transfected with an SV40 LT expression construct and selected in puromycin. Surviving cells were serially passaged to confirm immortalization.

Primary pig breast epithelial cells were isolated as described previously (Prather et al., 1999) and transfected with an SV40 LT expression vector. Transfected cells were isolated by selection in puromycin and surviving colonies pooled. As the cells were passaged, the SV40 LT transfected cells lost the senescent morphology apparent in the parental cells (Figure 1). They were then serially passaged to determine if they had been immortalized. Transfected cells have been passaged more than 26 times without apparent loss of viability. In contrast, parental cultures lose proliferative capacity by passage 8.

### Identification of an effective porcine BRCA1 miRNA

**Figure 2:**
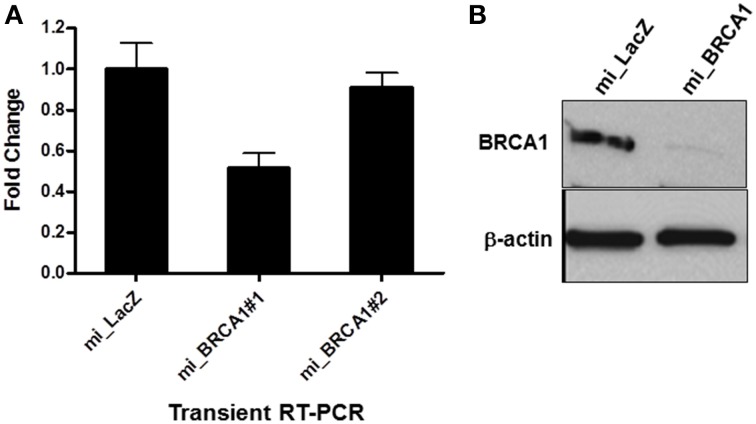
**miRNA-mediated BRCA1 knockdown in the immortalized pig mammary epithelial cells. (A)** The immortalized pig breast epithelial cells were transiently transfected with expression constructs for two BRCA1 miRNAs and a LacZ control. Forty-eight hours later, BRCA1 mRNA levels were determined by qRT-PCR analysis. **(B)** The immortalized pig mammary epithelial cells were transfected with BRCA1 miRNA#1 or the miLacZ control and selected with blasticidin to obtain cells that were stably knocked down for BRCA1. Western blot analysis confirmed efficient knockdown. β-actin served as control for equal protein loading. Error bars show standard error, *p* < 0.05 for miRNA#1, mRNA #2 was not significant.

The Block-iT™ RNAi Designer tool from Invitrogen was used to identify potentially effective miRNA sequences against porcine BRCA1. Two were generated and cloned into the vector pcDNA GW 6.2 EmGFPmiRNA. The vectors were then transiently transfected into the immortalized breast epithelial cells and assayed for the degree of knockdown by RT-PCR. Only one of the miRNAs proved effective (Figure 2A). This miRNA and the empty vector were stably transfected into the immortalized pig breast cells to generate a matched pair +∕− for BRCA1. Western analysis confirmed that the miRNA transfected cells had almost completely lost BRCA1 protein expression (Figure 2B).

### Suppression of BRCA1 enhances porcine epithelial cell growth

**Figure 3:**
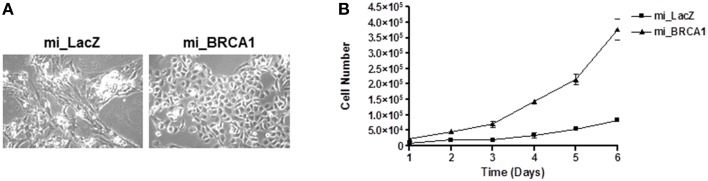
**Loss of BRCA1 enhances pig mammary epithelial cell growth. (A)** Serially passaging the pig mammary epithelial cells stably knocked down for BRCA1 resulted in an altered morphology compared to those cells stably expressing the LacZ miRNA. **(B)** 2 × 10^4^ cells/well were plated in 6-well plates and cell growth was determined by counting the number of cells at the indicated times. Error bars show standard error, *p* < 0.05.

As the cells were passaged, the BRCA1 suppressed cells progressively adopted a noticeably different morphology than the vector control cell line (Figure 3A). To characterize the effect of the BRCA1 suppression on the cell cycle, we measured the relative growth of the matched pair of cell lines transfected with vector or miBRCA1. Cells were plated and counted every day for 1 week. The BRCA1 suppressed cells exhibited an enhanced growth rate (Figure 3B).

### Suppression of BRCA1 alters differentiation

**Figure 4:**
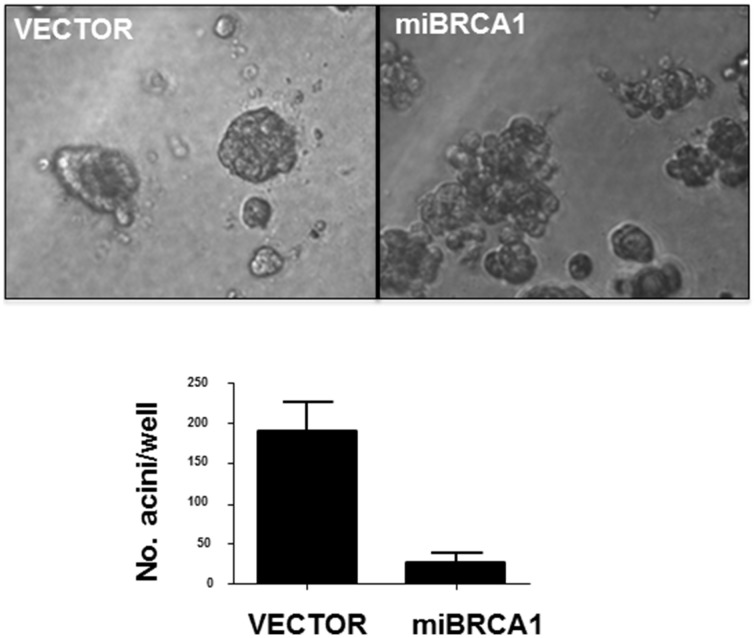
**Loss of BRCA1 inhibits acini formation**. The −∕+ BRCA1 pig mammary epithelial cells were plated in matrigel and allowed to grow for 10 days. Control cells formed acinus-like structures after 3D growth whereas the cells stably expressing BRCA1 miRNA grew as disordered masses. Error bars show standard error, *p* < 0.05.

Non-transformed human breast epithelial cell lines can be induced to differentiate into acini with hollow lumens when plated in 3D in matrigel. This differentiation is thought to mimic the process that occurs during the development of breast ducts. The process is disrupted by suppression of BRCA1 (Furuta et al., 2005). To examine the loss of BRCA1 in porcine cells on this process, we plated the BRCA1+∕− matched cell lines in matrigel for 10 days. After 10 days, the immortalized cells transfected with vector alone formed acini, reminiscent of human immortalized breast cells. The BRCA1 knockdown cells mostly grew as disordered masses (Figure 4).

### Suppression of BRCA1 promotes transformation

**Figure 5:**
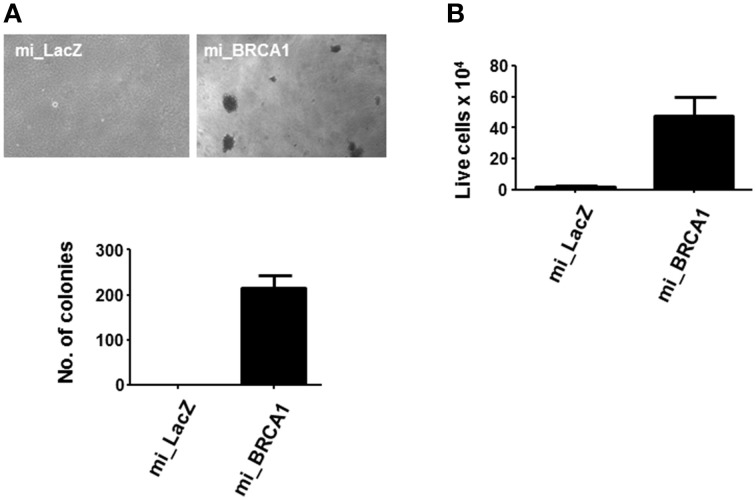
**Loss of BRCA1 enhances the transformed phenotype of pig mammary epithelial cells. (A)** The pig breast epithelial cells stably expressing BRCA1 miRNA were plated in soft agar and scored for growth 14 days later. Representative photomicrographs are shown in the top panel and data from three independent experiments quantitated in the bar graph in the lower panel. **(B)** 1 × 10^6^ cells/well were plated in polyHEMA-coated 12-well plates and cell viability assessed 48 h later by trypan blue staining. Error bars show standard error, *p* < 0.05.

The BRCA1 knockdown appeared to have induced enhanced growth and reduced differentiation (Figures 3, 4). In order to determine if it was sufficient to induce the tumorigenic phenotype, we plated the cells in soft agar and counted colony formation after 14 days. Anchorage-independent growth is one of the hallmarks of cell transformation and is considered the most accurate and stringent *in vitro* assay for detecting malignant transformation of cells (Colburn et al., 1978). Figure 5A shows that the BRCA1 positive cells failed to form colonies in agar. In contrast, the BRCA1 knockdown cells formed numerous, large colonies, indicative of highly transformed cells.

Suspension of normal cells results in the induction of apoptosis, a process called anoikis. Transformed cells typically resist anoikis, and this may contribute to their ability to proliferate when suspended in soft agar (Guadamillas et al., 2011). Examination of the ability of the cells to survive suspension showed that the BRCA1 knockdown cells were resistant (Figure 5B).

### BRCA1 knockdown promotes a CSC phenotype

**Figure 6:**
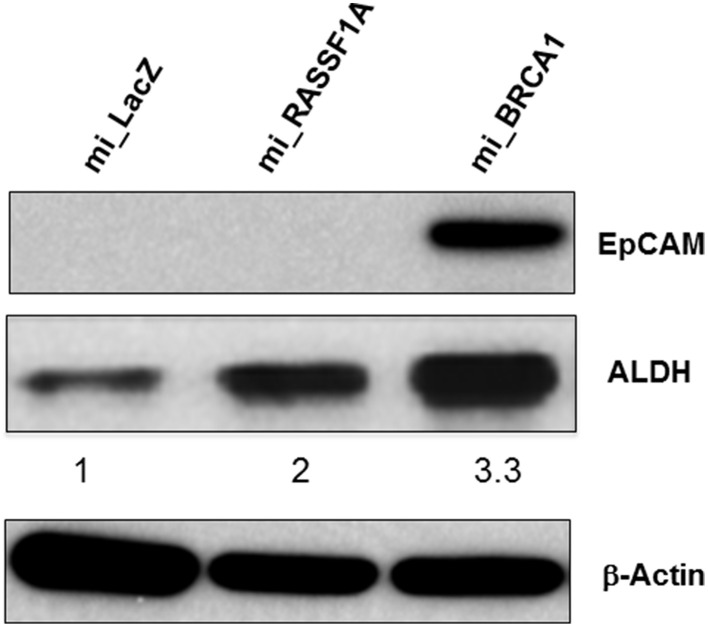
**BRCA1 knockdown in pig mammary epithelial cells alters CSC marker expression**. Equal amounts of protein lysates from control and BRCA1 knockdown cells were fractionated on SDS polyacrylamide gels and western blotted with an anti-EpCAM antibody and an ALDH1 antibody. β-actin was used a protein loading control. Mi_lacZ and a non BRCA1 miRNA transfected cell line (mi_RASSF1A) served as negative control cell lines.

In primary breast cells, knockdown of BRCA1 blocks the differentiation of stem/progenitor cells and enhances their proliferation (Furuta et al., 2005; Ma et al., 2010). Moreover, the ability to grow in soft agar is typically associated with the cancer stem cell (CSC) population of a transformed culture (Colburn et al., 1978). To determine if the knockdown of BRCA1 had promoted the development of CSC phenotype, we performed Western analysis for the expression of the CSC markers EpCAM (Dawood et al., 2014) as well as ALDH1 (Moreb, 2008). We found that in the BRCA1 knockdown cells, the EpCAM CSC marker was massively upregulated, and ALDH1 was upregulated three-fold (Figure 6). Actin served as a loading control. In these experiments, we had included miRNA against a second tumor suppressor, RASSF1A (Donninger et al., 2007), as an additional negative control. Whereas, the RASSF1A miRNA had no obvious effect on EpCAM, it did upregulate ALDH1, although less than the miBRCA1. Thus, RASSF1A may also be involved, to some extent, in CSC regulation.

## Discussion

Women who carry a BRCA1 germ line mutation have a cumulative lifetime risk of 50–85% of developing breast cancer (King et al., 2003). Although somatic BRCA1 mutations are rare in sporadic breast cancer, BRCA1 expression is down regulated in ~30% of sporadic cases (Yang et al., 2001). Its mode of action appears complex, subtle and remains only partially understood. It has been shown to modulate DNA repair, DNA damage checkpoints, stability of Claspin and Estrogen receptor alpha, and to modulate cell adhesion and motility (Wang, 2012; Christou and Kyriacou, 2013). Its loss of function in human cells is thought to promote genetic instability, hence leading to the development of cancer. It has been shown to synergize with the p53 tumor suppressor in mouse models and human cell tissue culture experiments (Brodie and Deng, 2001; Hartman and Ford, 2003).

Although mouse model systems have proven to be powerful tools in the investigation of the nature of cancer *in vivo*, they suffer from a major drawback. Murine cancer genetics is much simpler than that of humans. Murine cells are much easier to transform than human cell systems. Whereas, human cells require at least five genetic lesions to convert from a normal cell to a tumor cell, mouse cells can be induced to transform by just two oncogenic lesions (Rangarajan et al., 2004; Kendall et al., 2005). Thus, mouse models may prove inaccurate when trying to model human cancer. In contrast, porcine cancer genetics is very similar to human cells. Pig cells require five or more oncogenic mutations to undergo transformation, much like humans (Adam et al., 2007). Thus, a pig cancer model is more likely to accurately reflect the human condition.

Nothing is known about the role of BRCA1 in porcine cells and whether its ablation phenocopies the human state. Here, we have attempted to address the issue by generating the first immortalized porcine breast cell line by introducing an SV40 LT expression plasmid into primary breast cells derived from a pig. SV40 LT can immortalize human cells impairing the function of both the p53 and the Rb tumor suppressors (Ahuja et al., 2005). In experimental human cell systems, SV40 LT transduction has been shown to promote a transcriptional fingerprint which is quite reminiscent of that observed in triple negative breast cancer primary tumors (Deeb et al., 2007), suggesting the lesion is a relevant model. We found that it is also effective in a porcine system. We then examined the effects of inactivating BRCA1 in the immortalized cells.

To knockdown BRCA1, we used a stable miRNA expression approach. Although we assayed two different miRNA sequences, only one was really effective as measured by qRT-PCR, and so this is the sequence we used in the experiments. Subsequent examination of BRCA1 protein levels by Western blot showed that this miRNA rendered the BRCA1 protein almost undetectable. The knockdown of BRCA1 in a background where SV40LT has impaired p53 and Rb function was sufficient to promote enhanced growth and a dramatic transformation of the cells, as measured by colony formation in soft agar. Thus, we have created the first porcine breast epithelial tumor cell line.

BRCA1 down-regulation has been implicated in the development of a cancer stem cell-like phenotype in breast cells (Liu et al., 2008). *In vitro*, it appears that it is the CSC population that provides the ability to form colonies in soft agar (Colburn et al., 1978). When we examined the cells we found that the inactivation of BRCA1 in the SV40 LT background induced the upregulation of the CSC markers EpCAM (Munz et al., 2009) and ALDH1 (Moreb, 2008). This suggests that breast cancer CSC in humans and pigs are regulated in a similar manner by BRCA1.

This work establishes the first porcine model system for studying BRCA1 and breast cancer. It validates the concept that porcine transgenic animal models may be valuable for the study of human breast cancer and the development of novel therapeutics for the treatment of breast cancer driven by BRCA1 defects. In particular, due to the human-like life span of pigs, a porcine model of BRCA1 driven breast cancer could allow the testing of long term preventative measures, as well as strategies to counter the persistence of minimal residual disease after treatment. Attempts have been previously made to develop such an animal (Luo et al., 2011). Unfortunately, no animal's survived BRCA1 knockout long enough to determine any biological effects on breast cancer. These experiments suggest that a future porcine BRCA1 system would need to involve a tissue specific knockout, as has been the case in transgenic mouse systems.

### Conflict of interest statement

The authors declare that the research was conducted in the absence of any commercial or financial relationships that could be construed as a potential conflict of interest.
